# Differences in Blood Urea and Creatinine Concentrations in Earthed and Unearthed Subjects during Cycling Exercise and Recovery

**DOI:** 10.1155/2013/382643

**Published:** 2013-08-27

**Authors:** Paweł Sokal, Zbigniew Jastrzębski, Ewelina Jaskulska, Karol Sokal, Maria Jastrzębska, Łukasz Radzimiński, Robert Dargiewicz, Piotr Zieliński

**Affiliations:** ^1^Department of Neurosurgery, Military Clinical Hospital, Powstancow Warszawy 5, 85-681 Bydgoszcz, Poland; ^2^University of Physical Education and Sport, Gorskiego 1, 80-336 Gdansk, Poland; ^3^Novo-Med Non-Public Health Care Institution, Kurpinskiego 12/10, 85-096 Bydgoszcz, Poland; ^4^Medical University, Debinki 1, 80-211 Gdansk, Poland

## Abstract

Contact of humans with the earth, either directly (e.g., with bare feet) or using a metal conductor, changes their biochemical parameters. The effects of earthing during physical exercise are unknown. This study was carried out to evaluate selected biochemical parameters in subjects who were earthed during cycling. In a double-blind, crossover study, 42 participants were divided into two groups and earthed during exercise and recovery. One group was earthed in the first week during 30 minutes of cycling exercise and during recovery, and a second group was earthed in the second week. A double-blind technique was applied. Blood samples were obtained before each training session, after 15 and 30 minutes of exercise, and after 40 minutes of recovery. Significantly lower blood urea levels were observed in subjects earthed during exercise and relaxation. These significant differences were noted in both groups earthed at the beginning of exercise (*P* < 0.0001), after 15 (*P* < 0.0001) and 30 minutes (*P* < 0.0001) of exercise, and after 40 minutes of relaxation (*P* < 0.0001). Creatinine concentrations in earthed subjects during exercise were unchanged. *Conclusions*. Earthing during exercise lowers blood urea concentrations and may inhibit hepatic protein catabolism or increase renal urea excretion. Exertion under earthing may result in a positive protein balance.

## 1. Introduction

Direct contact of humans with the earth or using a metal conductor changes the electric potential on the surface of the body, as well as within the entire human organism. Transmitted earth potentials have a direct effect on the density of negative charges in the electrical human environment. Changes in the electrical potential of the aqueous, extracellular environment affect the modulation of bioelectrical processes. Up-and-down movement of the insulated human organism causes transient changes in potentials in the human electrical environment. During the same movement in the earthed human body, potentials in the electrical environment remain constant [1]. Differentials in charge influence biochemical processes. A change in the electrical environment alters the pH of biological fluids and the charge distribution on molecules, thereby affecting the function of enzymes and reactions, which are sensitive to alterations in pH. The ability of a substrate or enzyme to donate or accept hydrogen ions is influenced by pH [2, 3]. Therefore, earthing, which is direct contact with the earth of the human body or with the use of a metal conductor, affects human physiological processes. This effect is predominantly observed during night relaxation and during physical activity. Earthing during rest lowers blood concentrations of sodium, potassium, magnesium, iron, ionized calcium, inorganic phosphorus, and renal excretion of calcium and phosphates. The continuous earthing of the human body decreases blood glucose in patients with diabetes. Seven-hour earthing of an insulated human body causes a decrease in serum concentrations of total protein and albumin, while it increases the concentration of globulins. However 1-hour interruption of human contact with the earth causes an increase in total protein and globulin concentrations [4].

Exercise increases energy expenditure, resulting in promotion of amino acid catabolism, especially the oxidation of certain amino acids [5, 6]. Light to moderate exercise results in an increase in net protein catabolism and urea and creatinine excretion [7]. Foran et al. showed that short-term effects of exercise lead to an increase in glucose, total protein, albumin, uric acid, calcium, phosphorous, blood urea nitrogen (BUN), creatinine, total and direct bilirubin, alanine aminotransferase (ALT), aspartate aminotransferase (AST), and alkaline phosphatase levels 4 hours after marathon. BUN, creatinine, uric acid, ALT, AST, and direct bilirubin levels remained elevated 24 hours after the race, while glucose, total protein, albumin, globulin, calcium, phosphorous, total bilirubin, and alkaline phosphatase levels returned to baseline [8]. However, there are no reports on the effects of earthing during physical exercise. 

Therefore, we investigated biochemical changes in humans during exertion and recovery. We focused our investigation on blood urea and creatinine concentrations.

## 2. Materials and Methods

**Figure 1:**
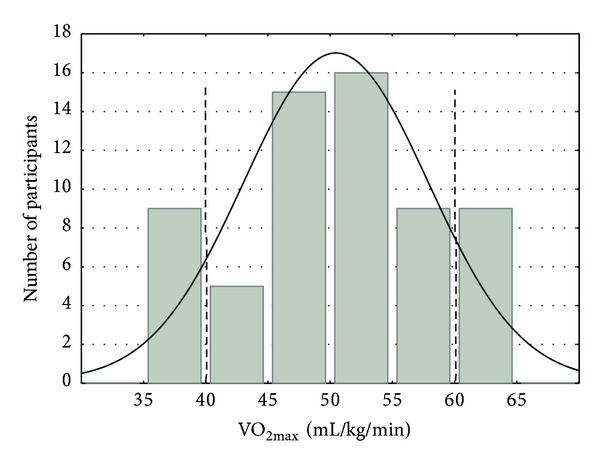
Distribution of VO_2max⁡_ (total number of measurements: 60).

Forty-two male volunteers were selected from a group of 60 students at the University of Physical Education and Sport in Gdansk, Poland. All of the participants were informed about details of the experiment and gave written consent. The ethics committee approved the investigation, which was conducted according to the principles expressed in the Declaration of Helsinki. Participants had no requirements or restrictions regarding their daily diet. Selection of the volunteers was based on an exertion test performed on a bicycle ergometer and on analysis of expired gases with the use of the Oxycon Pro analyzer (Jaeger, Wuerzburg, Germany). Volunteers were divided into two groups (A and B) based on maximal oxygen uptake (VO_2max⁡_) values. Subjects (*n* = 18) with the highest and lowest values of VO_2max⁡_ were excluded. Cut-offs were as follows: minimal VO_2max⁡_ = 40 mL/kg/min and maximal VO_2max⁡_ = 60 mL/kg/min (Figure 1). The other subjects (*n* = 42) were divided into two homogeneous groups consisting of 21 participants each (Table 1). The double-blind technique was applied. In the first week of the experiment, individuals from group A were earthed (A_0_) and those from group B were unearthed (B_1_). In the second week of the experiment, individuals from group A were unearthed (A_1_) and those from group B were earthed (B_0_). None of the participants knew if he was to be earthed in the first or the second week of the experiment. Tested persons had to perform two training exercises lasting 30 minutes on a bicycle ergometer once with earthing and the second time without earthing to the limit of 50% of VO_2max⁡_ (30-minute training exercise and 40 minutes of recovery).

**Figure 2:**
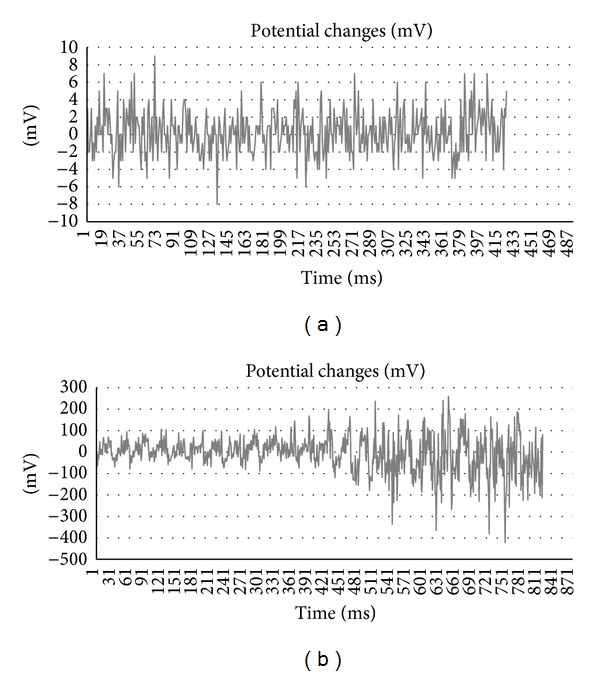
(a) Representative example of the potentials measured in an earthed subject during exercise. (b) Representative example of the potentials measured in an unearthed subject during exercise.

We measured the electrical potential of the body (Figures 2(a) and 2(b)) and blood parameters. Blood samples were obtained before each training session, after 15 minutes of exercise, after 30 minutes of exercise, and after 40 minutes of recovery. During training, continuous monitoring of physiological parameters was performed. Earthing was performed with the Pomona Electronics (USA) system consisting of four metal-plastic hypoallergenic bands wrapped around the ankle of the leg at the beginning of the trial. Bands were connected to conductors with a terminator clamp placed on plumbing pipe. All participants had wrapped bands around their ankles connected to a cable leading to a pipe through a switch, which enabled earthing to be turned off. None of the participants knew if he was connected or disconnected.

Biochemical analysis was conducted with the use of the A-15 analyzer (Biosystems SA, Costa-Brava, Barcelona, Spain). Urea concentrations were measured enzymatically with urease and glutamine dehydrogenase, and creatinine levels were determined with the kinetic calorimetric method with alkaline picrate.

Statistical analysis of the results was performed using repeated measures analysis of variance with a grouping variable followed by post hoc Fisher's least significant difference (LSD) test with alpha set at 0.05. The repeated measures factors were as follows: (1) earthing or the lack of earthing and (2) four different time points of measurement (rest, the 15th minute of exercise, the 30th minute of exercise, and the 40th minute of recovery). Grouping factors were in the order in which the subjects were earthed. Intergroup comparisons (earthed versus unearthed) were performed. A *P* value <0.05 was accepted as the level of statistical significance. To minimize the familywise probability of a type I error, only between-subject comparisons at the times 0, 15, 30, and 40 minutes were considered. All calculations were performed in STATISTICA version 10 (StatSoft, Inc., Tulsa, OK, USA).

## 3. Results

**Figure 3:**
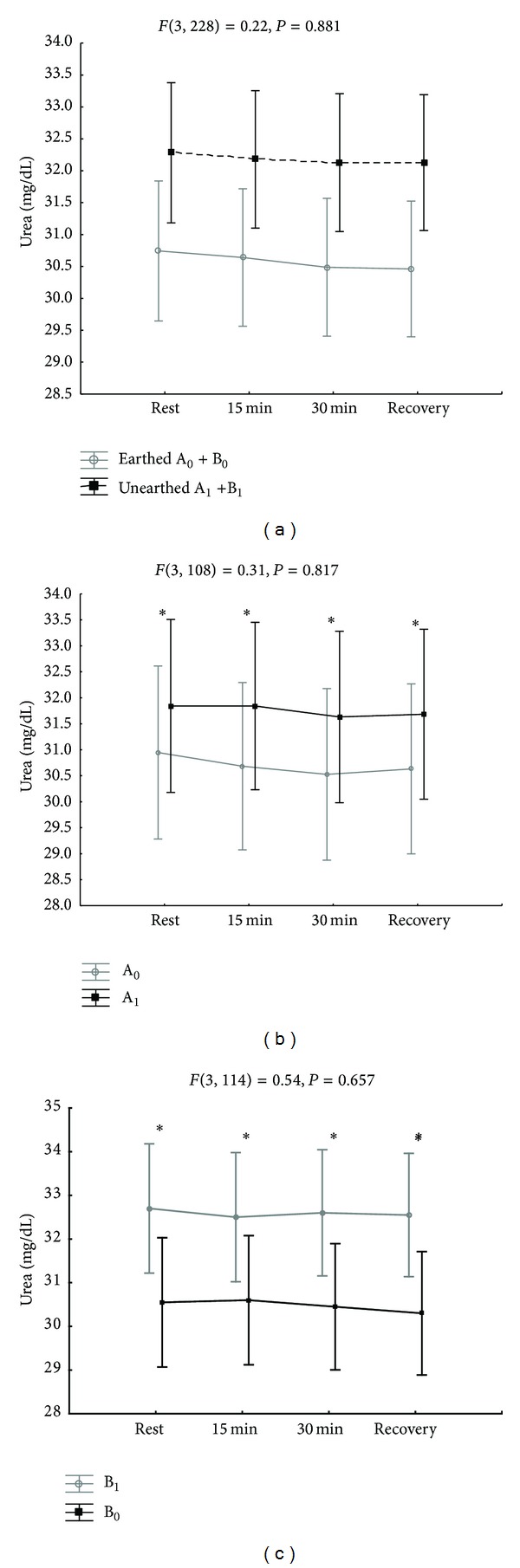
(a) Changes in blood urea levels in earthed (A_0_ + B_0_) and unearthed (A_1_ + B_1_) subjects during the first and second weeks of the experiment. (b) Changes in blood urea levels in earthed subjects in the first week of the experiment (A_0_) and unearthed subjects in the second week of the experiment (A_1_). *P < 0.001 between the groups. (c) Changes in blood urea levels in unearthed subjects (B_1_) in the first week of the experiment and earthed subjects in the second week of the experiment (B_0_). *P < 0.001 between the groups.

**Figure 4:**
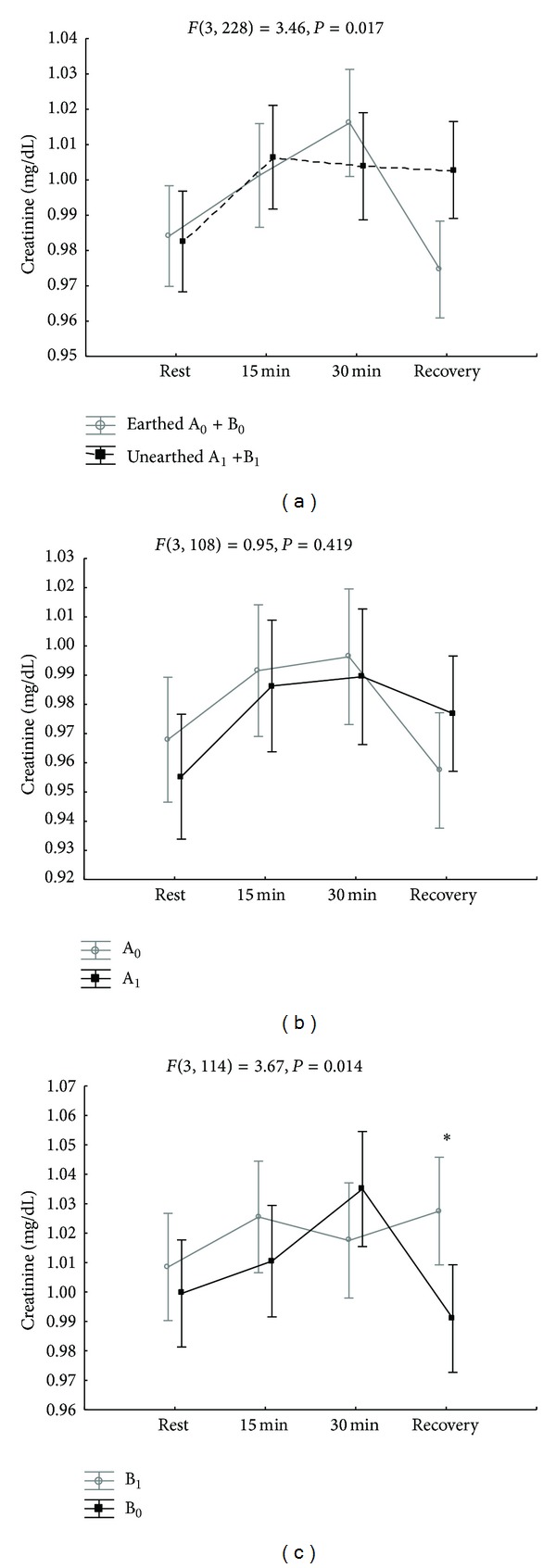
(a) Changes in blood creatinine levels in earthed subjects (A_0_ + B_0_) and unearthed subjects (A_1_ + B_1_) in the first and second weeks of the experiment. (b) Changes in blood creatinine levels in earthed subjects (A_0_) in the first week of the experiment and unearthed subjects (A_1_) in the second week of the experiment. (c) Changes in blood creatinine levels in unearthed subjects (B_1_) in the first week of the experiment and earthed subjects (B_0_) in the second week of the experiment. *P < 0.05 between the groups.

Significantly lower blood urea levels were observed in subjects earthed during exercise and relaxation (Table 2). There were significant differences in blood urea levels between subjects earthed in the first week and those unearthed in the second week (Figure 3(b)) and between subjects who were earthed in the second week and those unearthed in the first week (Figure 3(c)). These differences were clearer when considering the two groups, the earthed subjects and the unearthed subjects, regardless of the week in which they were earthed (Figure 3(a)). These significant differences in blood urea levels were observed in both groups at the beginning of exercise (*P* < 0.0001), during exercise after 15 minutes (*P* < 0.0001) and 30 minutes (*P* < 0.0001), and after 40 minutes of relaxation (*P* < 0.0001). Creatinine concentrations in earthed subjects were not significantly changed in the exercise phase but were significantly lower in the 40th minute of the recovery phase in earthed subjects in the second week (Table 3) (Figures 4(a), 4(b), and 4(c)).

## 4. Discussion

In our experiment, we selected a homogeneous group of young, healthy men with similar aerobic endurance measured by the indicator of maximal oxygen uptake. In all cases, we observed lower blood urea levels in individuals under earthing compared with unearthed subjects from the beginning to the end of the experiment during exertion and recovery.

Foran et al. showed elevated BUN and creatinine levels 4 hours after exercise as a result of dehydration and decreased renal perfusion [8]. In our study, we showed the opposite results to Foran et al.'s study [8] regarding blood urea concentrations. All subjects earthed during the exercise phase and in the recovery period had diminished levels of urea. Urea is a waste product of amino acid catabolism. Consequently, its plasma concentration is directly related to the amount of protein in the diet [9]. Our study showed that the observed alterations in blood urea levels were not dependent on a high or low protein diet. Urea is excreted by the kidneys. Urea is filtered by the glomerular capillaries, and it enters the renal tubule. Approximately half of urea is reabsorbed passively by diffusion, but the remainder is excreted in the urine. Lower blood urea levels suggest increased glomerular filtration and excretion in the urine or diminished reabsorption in the tubules. All these processes may result from changes in the electrical, aqueous environment in humans who are earthed. Urea is passively reabsorbed in the renal tubules. The rate of transport is determined by the electrochemical gradient for diffusion of the substance across the membrane and the permeability of the membrane for the substance. Additionally, glomerular filtration depends on the negative charge of the basement membrane of podocytes, which restrict large negatively charged molecules [10, 11]. Changes in the electrical potential of the membrane of tubular and glomerular cells can affect filtration and absorption.

Another waste product of metabolism is creatinine, which is a larger molecule than urea, and is impearmeant to the tubular membrane. Therefore, almost all of the creatinine filtered by the glomerulus is excreted in the urine [10, 11]. In our study, in contrast to the changes in urea, we did not observe altered levels of creatinine in the exercise phase. Lower creatinine concentrations at the end of the recovery phase in earthed subjects may have resulted from increased kidney filtration.

Urea formation occurs in the liver as a result of the process of deamination of amino acids and use of ammonia [12]. The activity of enzymes participating in the urea cycle is dependent on hydrogen ion concentrations, which can be affected by alterations in the distribution of charges in the aqueous environment during earthing. At a low pH, urea synthesis is decreased and HCO_3_^−^ consumption is reduced. HCO_3_^−^ and NH_4_^+^ are substrates for urea synthesis [13]. We consider that lower levels of urea in training subjects under earthing may be caused by decreased urea formation in the urea cycle. Therefore, earthing may inhibit hepatic catabolism of proteins during exercise. Excessive breakdown of proteins is observed in individuals during space flight and inactivity [10, 11]. In this aspect, earthing can have the opposite effect on metabolism of proteins. Resistance exercise increases muscle protein synthesis [10, 11]. Rates of muscle protein synthesis and degradation during exercise and in recovery are affected by the type of exercise, age, and state of nutrition. Exercise depresses muscle protein synthesis, whereas muscle protein breakdown probably remains unchanged during exercise. After exercise in the fasted state, synthesis and breakdown of proteins are elevated, when the net muscle protein balance remains negative [14]. A single bout of resistance-type exercise accelerates muscle protein synthesis rates. An increased rate of protein synthesis persists hours after exercise [15, 16]. Resistance exercise under earthing may multiply this effect. Direct contact with the earth or with a wire during exercise decreases blood urea nitrogen levels, which could result from the inhibition of breakdown of proteins in various tissues. However, there is no convincing evidence for reduced proteolysis of contractile proteins in active muscle in this study. At the end of the recovery phase, significantly lower creatinine levels suggest decreased breakdown of muscle creatine. A positive protein balance could be the result of earthing during exercise. Contact with the earth may have an important effect on human health in rest and exercise [17], especially for training athletes. Their goal is to maintain or increase lean body mass. An increase in muscle size and thus mass is caused by an increase in protein synthesis. Increased protein synthesis is reflected by a positive nitrogen balance. Earthing during exercise prevents protein degradation and thus helps to sustain a positive nitrogen balance.

## 5. Conclusion

Our study shows that blood urea concentrations are lower in subjects who are earthed (connected to the earth potential with the use of copper wire) during physical exercise and recovery compared with the same subjects who are not earthed during the same period of exercise and recovery. These results suggest that earthing during exercise inhibits hepatic protein catabolism or increases renal urea excretion. Earthing during exercise affects protein metabolism, resulting in a positive nitrogen balance. This phenomenon has fundamental importance in understanding human metabolic processes and may have implications in training programs for athletes.

## Figures and Tables

**Table 1:** Characteristics of participants in groups A and B.

	Group A (*n* = 21)	Group B (*n* = 21)
Age (years)	21.0 ± 1.00	21.1 ± 0.89
Weight (kg)	77.1 ± 10.05	72.8 ± 6.22
Height (cm)	183.6 ± 6.17	182.2 ± 6.15
VO_2_max	50.8 ± 4.17	50.7 ± 3.95

**Table 2:** Blood urea (mean ± SD) levels in mg/dL in volunteers in the first week (groups A_0_ (earthed) and B_1_ (unearthed)) and in the second week of the experiment (groups A_1_ (unearthed) and B_0_ (earthed)).

	I (rest)	II (15 min of exercise)	III (30 min of exercise)	IV (40 min of recovery)
A_0_	30.95 ± 7.34^∗I–III^	30.68 ± 6.84	30.53 ± 7.01	30.63 ± 7.06
A_1_	31.84 ± 7.17	31.84 ± 7.20	31.63 ± 7.37	31.68 ± 7.20
B_0_	30.55 ± 6.24	30.60 ± 6.17	30.45 ± 5.91	30.30 ± 5.79
B_1_	32.70 ± 6.98	32.50 ± 7.03	32.60 ± 7.00	32.55 ± 6.81

*Significant differences between phases at *P* < 0.05.

**Table 3:** Blood serum creatinine (mean ± SD) levels in mg/dL in volunteers in the first week (groups A_0_ (earthed) and B_1_ (unearthed)) and in the second week of the experiment (groups A_1_ (unearthed) and B_0_ (earthed)).

	I (rest)	II (15 min of exercise)	III (30 min of exercise)	IV (40 min of recovery)
A_0_	0.97 ± 0.09^∗I–III^	0.99 ± 0.11^∗II–IV^	1.0 ± 0.09^∗III-IV^	0.96 ± 0.08
A_1_	0.96 ± 0.09^∗I-II-III^	0.99 ± 0.09	0.99 ± 0.11	0.98 ± 0.09
B_0_	1.00 ± 0.08	1.01 ± 0.09	1.04 ± 0.09	0.99 ± 0.09
B_1_	1.01 ± 0.08^∗I–III^	1.03 ± 0.08	1.02 ± 0.09^∗III-IV^	1.03 ± 1.07

*Significant differences between phases at *P* < 0.05.
